# Stroke Induces Prolonged Changes in Lipid Metabolism, the Liver and Body Composition in Mice

**DOI:** 10.1007/s12975-019-00763-2

**Published:** 2019-12-21

**Authors:** Michael J. Haley, Claire S. White, Daisy Roberts, Kelly O’Toole, Catriona J. Cunningham, Jack Rivers-Auty, Conor O’Boyle, Conor Lane, Oliver Heaney, Stuart M. Allan, Catherine B. Lawrence

**Affiliations:** Division of Neuroscience and Experimental Psychology and Lydia Becker Institute of Immunology and Inflammation, Faculty of Biology, Medicine and Health, Manchester Academic Health Science Centre, The University of Manchester, Manchester, M13 9PT UK

**Keywords:** Brain ischaemia, High-fat diet, Adipokines, Liver, Lipids, Depression

## Abstract

During recovery, stroke patients are at risk of developing long-term complications that impact quality of life, including changes in body weight and composition, depression and anxiety, as well as an increased risk of subsequent vascular events. The aetiologies and time-course of these post-stroke complications have not been extensively studied and are poorly understood. Therefore, we assessed long-term changes in body composition, metabolic markers and behaviour after middle cerebral artery occlusion in mice. These outcomes were also studied in the context of obesity, a common stroke co-morbidity proposed to protect against post-stroke weight loss in patients. We found that stroke induced long-term changes in body composition, characterised by a sustained loss of fat mass with a recovery of lean weight loss. These global changes in response to stroke were accompanied by an altered lipid profile (increased plasma free fatty acids and triglycerides) and increased adipokine release at 60 days. After stroke, the liver also showed histological changes indicative of liver damage and a decrease in plasma alanine aminotransferase (ALT) was observed. Stroke induced depression and anxiety-like behaviours in mice, illustrated by deficits in exploration, nest building and burrowing behaviours. When initial infarct volumes were matched between mice with and without comorbid obesity, these outcomes were not drastically altered. Overall, we found that stroke induced long-term changes in depressive/anxiety-like behaviours, and changes in plasma lipids, adipokines and the liver that may impact negatively on future vascular health.

**Electronic supplementary material:**

The online version of this article (10.1007/s12975-019-00763-2) contains supplementary material, which is available to authorized users.

## Introduction

Stroke is the leading cause of long-term disability in the UK, chiefly due to the devastating effects of ischaemic brain damage on the sensorimotor system. However, patients also develop other complications that have a negative impact on recovery and reduce their quality of life. In the days, weeks and months after stroke, patients are at increased risk of infections and develop complications such as changes in body weight and appetite, depression, fatigue, anxiety and/or cognitive impairment [1–4]. Stroke patients are also at an increased risk for future vascular events [5, 6]. Very little is known about the underlying mechanisms involved in post-stroke complications but long-lasting changes in metabolism and energy balance are likely key [7].

Weight loss in stroke patients is common and an important determinant of outcome [8–10]. During the acute phase of stroke, the nutritional status of patients worsens, resulting in malnutrition that interferes with the recovery of patients’ activities of daily living [11, 12]. Approximately 20% of surviving patients may then develop tissue wasting (cachexia), characterised by loss of lean and fat tissue, with these patients showing poor functional recovery compared to nutritionally healthy patients [7]. Patients at high risk of malnutrition also have a high risk of mortality at 6 months post-stroke [13], and malnutrition and low body weight are both then risk factors for further stroke recurrence [14–17]. The reasons why patients experience weight loss after stroke are not entirely clear, but are thought to include reduction in food intake and increased catabolic drive. Stroke in mice can induce an acute whole body metabolic response that involves changes in lipids and adipokines in the liver, adipose tissue and plasma and which could impact on energy balance and weight control [18]. Adipokines such as resistin and adiponectin are released from adipose tissue and early changes in both are reported in mice after stroke, suggesting disturbances in metabolic status. Adiponectin, resistin and lipids have also been linked to increased stroke risk in people [19, 20]. However, whether these acute changes in lipolysis and adipokines seen in experimental stroke are long lasting is unknown, but prolonged alterations in these physiological systems could potentially impact future vascular health.

Conditions that affect metabolism, for example obesity and diabetes, increase the risk of stroke and are thus common co-morbidities found in stroke patients [21–24]. These conditions may also affect the recovery from stroke, for example obesity worsens acute experimental stroke outcome [25–28]. In contrast to clinical studies that monitor patient recovery over several months, most of these studies in comorbid animals have focused on acute time points (e.g. 24–72 h). In fact, some clinical studies suggest obesity may actually be beneficial for stroke recovery, the so-called obesity paradox, which leads to reduced mortality in obese patients [14, 29]. One proposed biological explanation for these epidemiological observations is that excess energy stores in obesity protect against post-stroke weight loss [30], thus preventing the harmful effects of malnutrition on post-stroke recovery. However, whether obesity affects weight loss, lipids and adipokine release, and neurological recovery and behaviour after experimental stroke in the long-term is poorly understood.

The aims of this study were to (i) establish the effect of experimental stroke on depressive- and anxiety-like behaviours; long-term secondary complications commonly reported in stroke patients and (ii) assess if stroke-induced prolonged changes in weight loss, and adipokine and lipid status. Additionally, we determined the impact of obesity on these outcomes.

## Methods

### Animals

Male C57BL/6J mice (Envigo, UK) were used for all studies. All animals were housed in individually ventilated cages in standard housing conditions (temperature 21 ± 2 °C; humidity 55% ± 5%; 12-h light/12-h dark cycle), with access to food and water ad libitum. At 8 weeks of age, cages of mice were randomly assigned (using ‘rand()’ function of Microsoft Excel) to either a high-fat diet (named obese; 60% energy from fat, 58G9, Test Diets®, supplied by IPS Product Supplies Ltd., UK) or a low-fat diet (named control; 12% energy from fat, 58G7, Test Diets®, IPS Product Supplies Ltd., UK) for 26 weeks prior to surgery [25]. All experiments were conducted in accordance with the UK Animals (Scientific Procedures) Act 1986 and approved by the local Animal Welfare and Ethical Review Board, University of Manchester, UK. All reporting of animal experiments complied with the ARRIVE guidelines (Animal Research: Reporting in In Vivo Experiments) [31].

### Transient Middle Cerebral Artery Occlusion

Transient middle cerebral artery occlusion (MCAO) was used to induce focal ischemia in the left cerebral hemisphere using a protocol adapted from Longa et al. [32]. Anaesthesia was induced with 4% isoflurane and maintained with 1.5% (30% O_2_ and 70% N_2_O). Core body temperature was monitored using a rectal probe and maintained at 37 ± 0.5 °C with a homeothermic blanket (Harvard Apparatus, UK). The left carotid arteries were exposed and a silicone-coated filament (coating 210 μm in diameter and 4–5-mm length, Doccol, USA) was introduced into the external carotid artery and advanced along the internal carotid artery until occluding the origin of the MCA. Successful MCAO was confirmed by a reduction in cerebral blood flow of at least 80% as measured using laser-Doppler (Moor Instruments, UK). High-fat diet (for 6 months) is known to worsen ischaemic damage in rodents after 30 min but not 20 min MCAO [25]. As the severity of many outcome measures (e.g. post-stroke behavioural deficits) are likely to correlate with infarct volume, the greater ischaemic damage seen after 30 min MCAO in obese high-fat fed mice could be a potential confounding factor. Therefore, to produce a similar volume of ischaemic damage between diet groups, the MCA was occluded for 20 min in obese mice (*n* = 10) and 30 min in control mice (*n* = 19). To confirm that obese mice have poorer outcome (as previously reported [25]), a group of obese mice (*n* = 4) also underwent MCA for 30 min. After the respective occlusion times, the filament was removed to allow reperfusion and the wound sutured. Sham-operated mice (*n* = 10/diet group) underwent the same procedure; however, once the filament had been inserted and advanced to the MCA, it was immediately withdrawn. As we aimed to match infarct volumes between diet groups and did not have a pilot data for animals recovered to 60 days, we had no pilot data on which to base power calculations. Therefore, a standardised effect size was used for the power analysis, using a Cohen’s d of 1.4. Using an alpha of 0.5 and a power of 0.8, we found 10 animals per group would be required. However, additional animals were used in anticipation of drop-out over the 60-day recovery period due to the large infarcts expected. Due to the obese phenotype, blinding could not be performed during surgery, but all subsequent analyses were performed blinded to diet and surgery. To facilitate nutrition for the first 2 days post-MCAO, all animals were given access to a soft chow diet (BK001 E, Special Diets Services, UK), in addition to their respective control and high-fat diets. Deficits in sensorimotor function were assessed using a 28-point neuroscore on days 2, 7, 14 and 51 post-MCAO, modified from procedures described previously [33].

Due to animals reaching humane endpoints for animal suffering [34], several animals were euthanised within 14 days of surgery, some of which were euthanised prior to undergoing magnetic resonance imaging (MRI) imaging: 8 control (of which 3 had no MRI scan on day 2), 5 obese mice (20 min MCAO) and 4 obese mice (30 min MCAO). One animal each from the control 30 min and obese 20 min groups were excluded due to no stroke. No deaths or complications occurred in sham-operated animals. Final numbers were control sham *n* = 10, control MCAO/stroke *n* = 10, obese sham *n* = 10 and obese MCAO/stroke (20 min) *n* = 4 and are detailed in the figure legends.

### Quantification of Infarct Volume/Oedema/Brain Atrophy

Infarct volume and oedema were quantified at 48 h post-MCAO, and brain atrophy at day 50 post-surgery by MRI. Under isoflurane anaesthesia, MRI scans were taken with a 7T horizontal bore magnet (Agilent Technologies, UK) interfaced to a BrukerAvance III console (Bruker Biospin, UK) using a surface transmit-receive coil. Coronal pilot images were acquired to determine the correct geometry and localise the brain (using a multi-scale gradient echo sequence). T2-weighted TurboRARE high resolution images were taken under the following parameters: matrix = 256 × 256, slice thickness = 1 mm, interslice distance = 1 mm, resolution = 0.0156 cm/pixel, acquisition time = 5 min 51 s. Infarct volume at 48 h was calculated by measuring infarct area over 8 slices in ImageJ (NIH, USA). Oedema was calculated as the percentage difference between the volumes of the ipsilateral and contralateral hemispheres. At day 50, atrophy was calculated as the percentage reduction in the volume of the ipsilateral hemisphere compared to the contralateral. Analyses of atrophy, infarct volume and oedema were all performed blinded to experimental groups by randomising file names.

### Behavioural Phenotyping

Depressive-like behaviours were assessed in all mice using the burrowing and nest building tests. For all tests, a baseline measurement (day 0) was taken prior to surgery. Locomotor activity and measures of anxiety were assessed using the open field test. White noise was played throughout the tests to minimise auditory cues, and all testing (except nest building) was conducted during the light phase.

#### Burrowing

Burrowing ability was assessed in all mice on days 0, 3, 14 and 30 as adapted from Deacon et al. [35]. Briefly, burrows were made from 20 cm piece of 68-mm diameter plastic downpipe with one end sealed, 2.5-cm machine screws were inserted at the other end to elevate the burrow 3 cm off the cage floor. Mice were habituated to the burrowing arena (an empty cage) for 30 min, before burrowing tubes, containing 700 g of gravel, were introduced and left for 1 h. After 1 h, burrows were removed and remaining gravel was weighed to calculate percentage burrowed.

#### Nest Building

Nest building ability was assessed in all mice on days 0, 3, 7, 14 and 30. Mice were individually housed with 140 g of wood chippings and 20 g of sizzle nest building material equally distributed throughout the cage, and food weighed. Mice were left overnight and images were taken of the nests from above and of the sides to determine depth and all mice returned to home cages. Nests were scored based on Deacon’s standardized scale [36], from 0 to 5. All images were blinded and scored by two independent markers, an average score was calculated and further analysed. The amount of food consumed (kCal) during the dark phase was also calculated.

#### Open Field

On day 37, mice were placed individually into the centre of a square opaque perspex box (45 cm × 30 cm × 45 cm) and their behaviour recorded for 5 min. Apparatus was cleaned with 70% ethanol between mice. Video recordings were analysed with Stoelting ANY-maze v4.9 software and for assessment of locomotion/activity the total distance (m) moved, average speed (m/s), duration (sec) of mobile episodes were measured. For assessment of anxiety, the time (sec) spent at the sides of the arena was measured and % time spent at the sides calculated.

### Body Composition Analysis

Body composition (adipose tissue and lean mass) was assessed by nuclear magnetic resonance imaging (EchoMRI, Echo Medical systems, USA) at days 0, 1, 3, 5, 7, 10, 14, 21, 42 and 49. Total body weight of animals was taken prior to scanning. Net loss (g) in total body weight and adipose and lean mass was calculated from day 0 (prior to stroke) and area under the curve (g × days) for all time points.

### Tissue Preparation and Histology

At day 60, post-surgery mice were terminally anaesthetised with 4–5% isoflurane (30% O_2_ and 70% N_2_O) and cardiac blood taken before transcardial perfusion with phosphate buffered saline (PBS). Plasma was obtained by centrifugation at 1200*g* for 10 min and epididymal fat removed and both were frozen and stored at − 80 °C. Animals were then transcardially perfused with 4% paraformaldehyde (PFA) and the brain and liver were immerse fixed in PFA for 48 h. The brain was sliced into 2-mm-thick sections using a metal brain matrix. Brain sections and the liver were embedded in paraffin wax using a Shadon Citadel 2000 tissue processor (Thermo Scientific, UK) and subsequently sectioned into 5 μm sections using a Leica RM 2155 Microtome (Leica Microsystems Ltd., Germany) and mounted on SuperFrost© Plus slides (Thermo Scientific, UK). Brain and livers were deparaffinised in xylene (followed by ethanol), stained with haemotaoxylin and eosin and coverslipped using DPX mounting medium (Fisher Scientific, UK). Hepatic steatosis (fatty vacuolation due to accumulation of lipid) and hepatocyte injury (ballooning degeneration and/or pale to clear cytoplasm) were semi-quantitatively graded using the criteria adapted from that previously described by Kleiner et al. [37]. For each parameter, six random fields of view were evaluated using ImageJ by an experimenter blinded to the groups by randomising file names.

### Adipokine Analysis

Adipose tissue (from frozen epididymal fat) was homogenised in buffer (50 mM Tris-HCl, 150 mM NaCl, 5 mM CaCl_2_ and 0.02% NaN_3_) containing 1% Triton-X and protease inhibitor cocktail 1 (Calbiochem, UK). Five microlitres was added per milligram of tissue sample (around 100 mg of tissue was used). Samples were then homogenised using a T-10 Basic ULTRA-TURRAX homogeniser (IKA, USA), briefly sonicated on ice with a hand-held probe sonicator (IKA) and left on ice for 30 min. Samples were centrifuged at 14,000*g* for 30 mins (4 °C). Supernatant was decanted and stored at − 20 °C. Resistin, adiponectin and leptin in adipose tissue supernatant and plasma were analysed by enzyme-linked immunosorbent assay (ELISA; R&D Systems, UK) according to the manufacturer’s instructions. Adipokine concentrations were determined by reference to the relevant standard curves. For adipose tissue, protein concentration was assessed by a bicinchoninic protein assay (BCA; Pierce Biotechnology, USA), and results expressed as pg or ng/mg protein. For plasma, data are expressed as μg or ng/ml/g fat weight.

### Lipid Analysis and ALT Assay

Plasma levels of free fatty acids (FFAs; Zen-Bio Inc., USA) and triglycerides (BioVision Inc., USA) were measured (in mmol/l) using the relative assays according to the manufacturer’s instructions. Alanine aminotransferase (ALT) levels were measured in the plasma using a colorimetric activity assay kit (Cayman Chemical, USA).

### Data and Statistical Analyses

Data are presented as mean ± standard deviation (SD), unless indicated. Equal variance and normality were assessed with the Brown–Forsythe and Shapiro–Wilk test respectively and appropriate transformations were applied when necessary. For discrete data and data with non-normal distributions (neuroscore), generalised linear mixed modelling was used (GLMM) with mouse I.D. as the random effect [38–40]. The significance of inclusion of an independent variable or interaction terms was evaluated using log-likelihood ratio. Holm–Šidák post hocs were then performed for pair-wise comparisons using the least square means [41]. Pearson residuals were evaluated graphically using predicted vs level plots. All analyses were performed using R (version 3.5.1) [42]. Linear mixed effects models were used to assess the effects of diet and stroke on depressive-like behaviour. The linear mixed model function “lme” (nlme v1.1-9) was used to estimate the effects of stroke and diet on outcome measures. All factors and interactions were modelled as fixed effects. A within-subject design with random intercepts and by-subject random slopes for all effects was used, aiming for a maximal random effects structure. Inclusion of fixed effect into the model was evaluated using the log-likelihood ratio test. Modelling was carried out using R (version 3.2.5). All other statistical analyses were performed using GraphPad Prism v6 (GraphPad Software Inc., USA) using the appropriate tests (as detailed in the figure legends). *p* values < 0.05 were considered statistically significant.

## Results

### Obese Mice with the Same Infarct Volume Showed No Difference in Acute Outcome

**Fig. 1:**
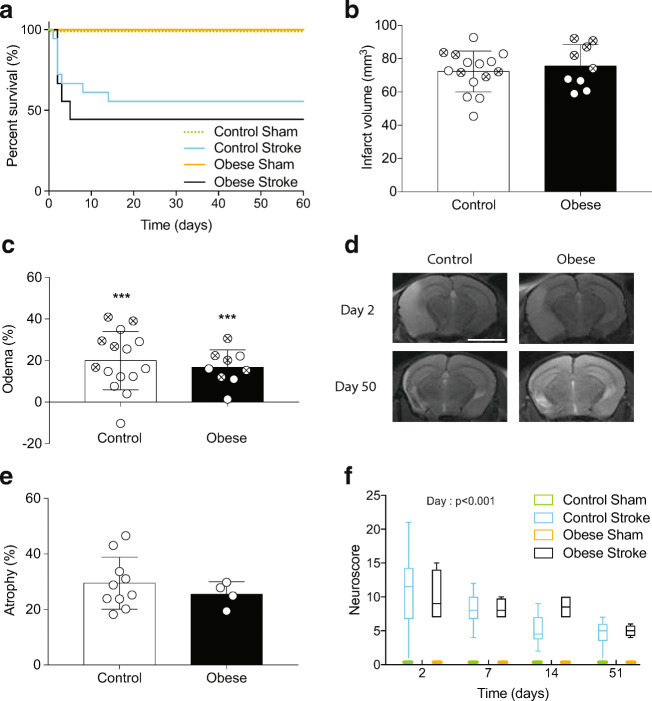
Obese mice with the same infarct volume showed no difference in outcome. C57BL/6J fed a control or high-fat (obese) diet for 6 months underwent middle cerebral artery occlusion (MCAO; 30 min for control, 20 min for obese). Percent survival over 60 days (**a**). MRI imaging (T2 Turborare) was performed on all mice at day 2, and in survivors at day 50. Infarct volume (**b**) and oedema (**c**) were quantified on day 2 using MRI images (crossed symbols represent mice culled prior to day 50). Representative MRI scans for both control and obese mice illustrating ischemic damage on day 2 and ipsilateral hemisphere atrophy on day 50 (**d**). Representative images for both time points are from the same mouse (scale bar = 0.38 cm). Atrophy in ipsilateral hemisphere was measured at day 50 from MRI scans (**e**). Neuroscore was measured at days 2, 7, 14 and 51 after surgery to assess sensorimotor deficits (**f**). Data are presented as mean ± SD (**a**, **b**, **c**, **e**) or median and interquartile range (**f**). For (**b**) and (**c**) *n* = 9–15 and (**e**) and (**f**) *n* = 4–10. Data were assessed using (**a**) a Log-rank (Mantel-Cox) test, (**b**) unpaired *t* test, (**c**) and (**e**) one-sample *t* test with hypothetical value of 0 within a group (where **p < 0.01, ***p < 0.001) and unpaired *t* test between groups, (**f**) generalised linear mixed effects modelling followed by Holm–Šidák post hoc analysis

Although obesity is a well-established risk factor for ischaemic stroke, it is more controversial as to whether obesity worsens outcome in patients. We have shown previously that with an occlusion time of 30 min, obesity (induced by high-fat feeding) increases ischaemic damage after experimental stroke in mice [25] and here, 30-min occlusion of the MCA also produced severe ischaemic damage and all mice (*n* = 4) had to be culled at day 2. To better model the clinical situation and avoid the confounding effect of differences in infarct volumes between groups, the MCA was occluded for 20 min in obese mice, compared to 30 min in control mice. There was no effect of sham surgery on survival in either diet group. There was no significant difference in survival between control mice undergoing a 30-min occlusion, and obese mice undergoing a 20-min occlusion (Fig. 1a). There was also no significant difference in infarct volume or stroke-induced oedema between groups as measured by MRI at day 2, either in all mice including those that died, or in survivors only (Fig. 1b, c).

MRI imaging at day 2 (Fig. 1d) showed large infarcts present in both the striatum and cortex, and in some cases extending into the hippocampus and thalamus. At day 50, areas of hyperintensity on MRI were present predominantly in the outer cortical regions. On histological examination, these regions were cerebral spinal fluid (CSF)-filled cavities devoid of brain tissue. Ventricular enlargement and ipsilateral hemisphere atrophy were also observed in both control and obese stroked animals, with no significant difference in atrophy between groups (Fig. 1e).

Sensorimotor function was assessed longitudinally using neuroscore. Deficits in both groups were observed up to 51 days post-stroke compared to sham animals (Fig. 1f). A significant effect of day (*p* < 0.001) was observed after stroke in control and obese mice indicating functional improvement (a reduction in neuroscore) over time. There was no effect of diet on neuroscore, and no interaction effect between diet and day.

### Stroke Disrupts Spontaneous Behaviours in Mice

**Fig. 2:**
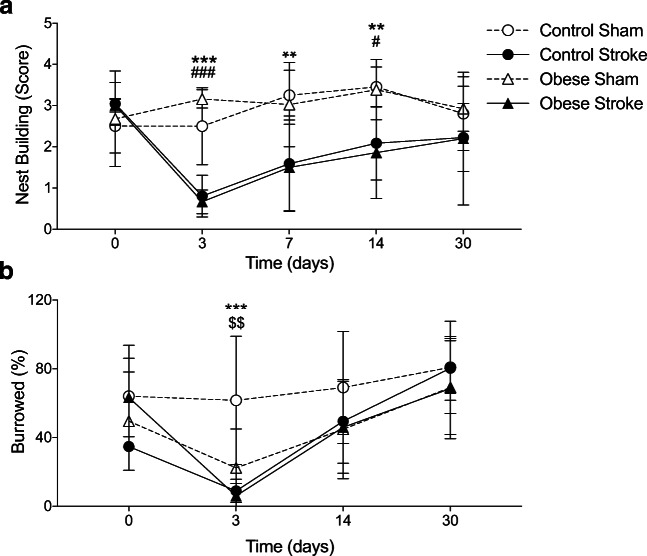
Stroke induces prolonged depressive-like behaviours. Depressive-like behaviours were assessed by nest building (**a**) and burrowing (**b**) on days: 0, 3, 7, 14 and 30 (not day 7 for burrowing) post-stroke. Impaired nest building behaviour in control and obese mice was observed up to 14 days after stroke, and at day 3 for burrowing, when obese sham mice also showed a deficit. Data are presented as mean ± SD (*n* = 4–10). **p < 0.01 and ***p < 0.001 control sham versus control stroke; ^#^*p* < 0.05 and ^###^*p* < 0.001 obese sham versus obese stroke; ^$$^*p* < 0.01 control sham versus obese sham. Statistical analysis was performed using a linear mixed effects models followed by Šidák–Holmes post hoc analysis

Both nest building and burrowing are behaviours engaged by healthy mice without training, and so the loss of these behaviours can be used as indication of depressive-like behaviour. Stroke induced deficits in both nest building (Fig. 2a) and burrowing (Fig. 2b). The stroke-induced deficit in nest building was detectable up to 14 days post-stroke, with a maximal reduction seen at 3 days. Mixed linear model analysis identified a main effect of stroke (${\upchi}_1^2$=7.076, *p* < 0.008), but no effect of diet, and no interaction between stroke and diet. Deficits in burrowing behaviour were observed at 3 days post-stroke compared to sham-operated control mice. However, sham-operated obese mice also had significantly reduced burrowing behaviour at day 3. Mixed linear model analysis identified a main effect of both diet (${\upchi}_1^2$=5.094, *p* < 0.024) and stroke (${\upchi}_1^2$=4.563, *p* < 0.033). An interaction effect between stroke and diet was observed (${\upchi}_1^2$=5.011, *p* < 0.025), and between stroke and day (${\upchi}_3^2$=13.284, *p* < 0.004).

**Fig. 3:**
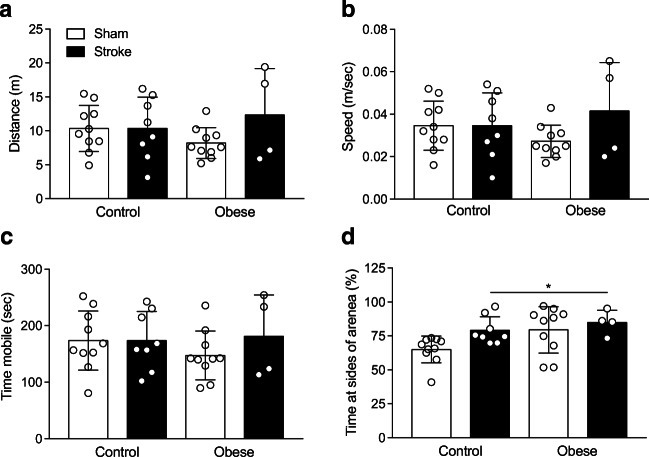
Stroke induces behaviours indicative of anxiety. Locomotor behaviour and measures of anxiety were assessed in the open field test on day 37 and expressed for locomotion as (**a**) total distance moved, (**b**) average speed, (**c**) time mobile, and for anxiety (**d**) percentage of time spent at sides of arena. An increase in the amount of time spent at the sides of the arena, indicating anxiety, was seen after stroke in control and obese mice and also in obese mice after sham surgery. Data are presented as mean ± SD (*n* = 4–10). *p < 0.05 for a main effect of stroke and diet. Statistical analysis was performed using a two-way ANOVA followed by Tukey’s post hoc multiple comparisons test

To determine if there were any changes in locomotion or anxiety-like behaviours, open field analysis was performed on day 37. No effect of diet or stroke was detected on the total distance travelled, average speed or time mobile (Fig. 3a–c). A significant main effect of diet and stroke was observed on measures of anxiety (time spent in the sides of arena), with stroke increasing anxious behaviour (Fig. 3d). No interaction effect between diet and stroke was observed on any parameters assessed. To assess working memory the Y-maze test was performed at day 14 and 45. No memory impairment was observed after stroke in either control or obese mice compared to sham (Fig. S1).

### Greater and Prolonged Adipose Tissue Loss in Obese Mice After Stroke

**Fig. 4:**
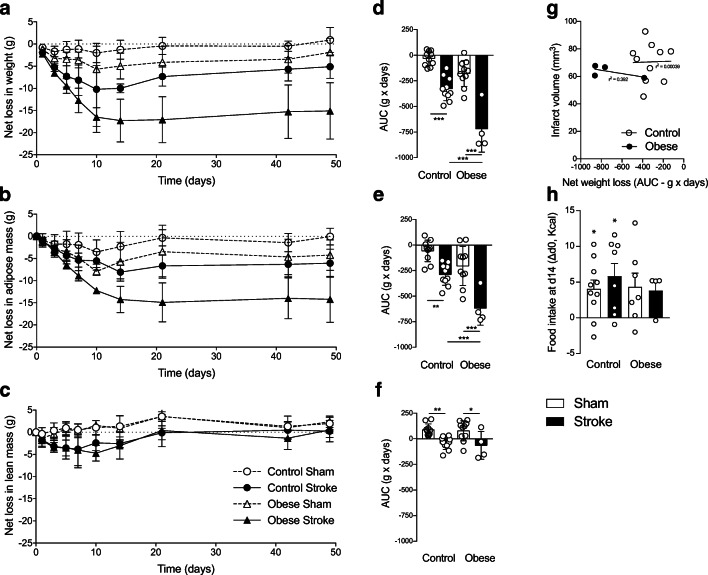
Stroke induced a prolonged reduction in adipose tissue mass. Net change in body weight (**a**), adipose/fat mass (**b**) and lean mass (**c**) in grams were measured at baseline and days 1, 3, 5, 7, 10, 14, 21, 42 and 49 after stroke. Fat and lean masses were determined by EchoMRI. Area under the curve was calculated for net change (**d–f**). A prolonged decrease in body weight due to loss of fat mass was observed in control and obese mice after stroke with greater loss in obese mice. Stroke induced a transient loss of lean mass in control and obese mice. No correlation was seen between body weight loss and infarct volume in control or obese mice (**g**). Overnight food intake on day 14 relative to food intake prior to surgery (day 0) (**h**) was significantly increased in control but not obese groups. Data are shown as mean values ± SD (*n* = 4–10/group). For AUC data, all groups were compared with a two-way ANOVA followed by Tukey’s post hoc multiple comparisons test, where *p < 0.05, **p < 0.01, ***p < 0.001. Difference in food intake was compared to a hypothetical mean of 0 by one-sample *t* test (where *p < 0.05). Correlation in (**g**) was performed using a linear regression

Before surgery (MCAO or sham), mice fed a high-fat diet had increased body weight and a higher percentage body fat but reduced lean mass (Table 1) compared to mice fed a control diet. After experimental stroke, both control and obese mice showed a prolonged loss of body weight compared to sham-operated mice (Fig. 4a, d, Table 1). However, body weight loss was significantly greater in obese mice. In both groups, post-stroke weight loss was primarily due to a reduction in adipose tissue mass, with obese mice losing significantly more adipose tissue (Fig. 4b, e). In comparison, lean mass reduced only transiently in both stroke groups, and the extent of the loss was similar between control and obese mice (Fig. 4c, f). In neither group did infarct volume correlate with weight loss (Fig. 4g). At day 14, when weight loss reached a nadir after stroke, overnight food intake was significantly greater compared to baseline (day 0) in both control-fed sham and stroke mice, but no difference was seen between day 0 and 14 in obese mice (Fig. 4h).

**Table 1:** Body composition before and after stroke

	Control		Obese	
	Sham	Stroke	Sham	Stroke
Initial				
Body weight	34.5 ± 5.8	34.0 ± 1.9	49.4 ± 1.7^###^	50.1 ± 3.9^###^
% fat	27.6 ± 13.2	28.5 ± 4.0	43.2 ± 2.2^###^	43.1 ± 3.2^#^
% lean	67.6 ± 12.2	67.3 ± 2.9	53.9 ± 2.2^###^	52.6 ± 1.9^##^
Final				
Body weight	35.4 ± 5.7	28.9 ± 1.5*	47.6 ± 4.4^###^	34.9 ± 8.5***
% fat	27.7 ± 11.0	11.3 ± 5.0***	35.6 ± 5.3	19.5 ± 8.9**
% lean	70.9 ± 8.7	79.9 ± 8.7	61.3 ± 4.5^#^	78.5 ± 9.3**

Mice were kept on a control diet or a high-fat diet (obese) for 6 months. Middle cerebral artery occlusion (MCAO; 30 min for control and 20 min for obese) to induce stroke, or sham surgery was performed. Body weight, and % fat and lean tissue were assessed before and at day 50 after surgery by EchoMRI. Data are shown as mean values ± S.D. (*n* = 4–10/group). Statistical analysis was performed using a two-way ANOVA followed by Tukey’s post hoc multiple comparisons test. *p < 0.05, **p < 0.01, ***p < 0.001 versus surgery control on same diet and ^#^*p* < 0.05, ^##^*p* < 0.01, ^###^*p* < 0.001 versus control diet for same surgery

### Stroke Induces a Prolonged Change in Adipokine Production

**Fig. 5:**
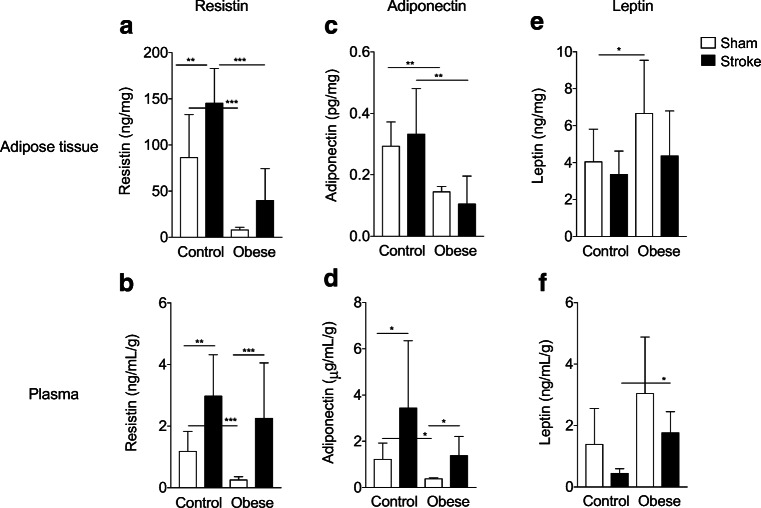
Stroke induces a prolonged change in adipokine production. White adipose tissue (epididymal, **a**, **c**, **e**) and plasma (**b**, **d**, **f**) was taken 60 days post-stroke and resistin (**a–b**), adiponectin (**c–d**) and leptin (**e–f**) levels assessed by ELISA. In control mice, stroke caused an increase in resistin in the adipose tissue and plasma, and an increase in plasma adiponectin. An increase in plasma resistin and adiponectin was also seen after stroke in obese mice. Adipose tissue concentrations were normalised to microgram of total protein from BCA assay and plasma concentrations normalised to total adipose mass. Data are presented as mean ± SD (*n* = 4–10). *p < 0.05, **p < 0.01 and ***p < 0.001. Statistical analysis was performed using two-way ANOVA followed by Tukey’s post hoc multiple comparisons test

In mice fed a control diet, stroke induced significant alterations in adipokine production at 60 days post-stroke (Fig. 5). Resistin concentrations were significantly increased after stroke in the adipose tissue (Fig. 5a) and plasma (Fig. 5b), and adiponectin concentrations were increased in the plasma (Fig. 5c). Leptin concentrations were unaffected by stroke (Fig. 5c, f). The adipokine response to stroke was similar in obese mice, though some differential effects were observed. For example, stroke did not induce a significant increase in resistin in the adipose tissue in obese mice. There was no significant correlation between the extent of weight loss and plasma adipokine levels after stroke in control or obese mice (adiponectin; control *r*^2^ = 0.09, obese *r*^2^ = 0.32: resistin; control *r*^2^ = 0.19, obese *r*^2^ = 0.29: leptin; control *r*^2^ = 0.0002, obese *r*^2^ = 0.35). Obesity by itself (in the absence of stroke) also affected adipokine production. Sham-operated obese mice had decreased resistin and adiponectin concentrations in the adipose tissue and plasma, and increased concentrations of leptin in adipose tissue.

### Stroke Induces a Prolonged Change in Plasma Lipids and Liver Function

**Fig. 6:**
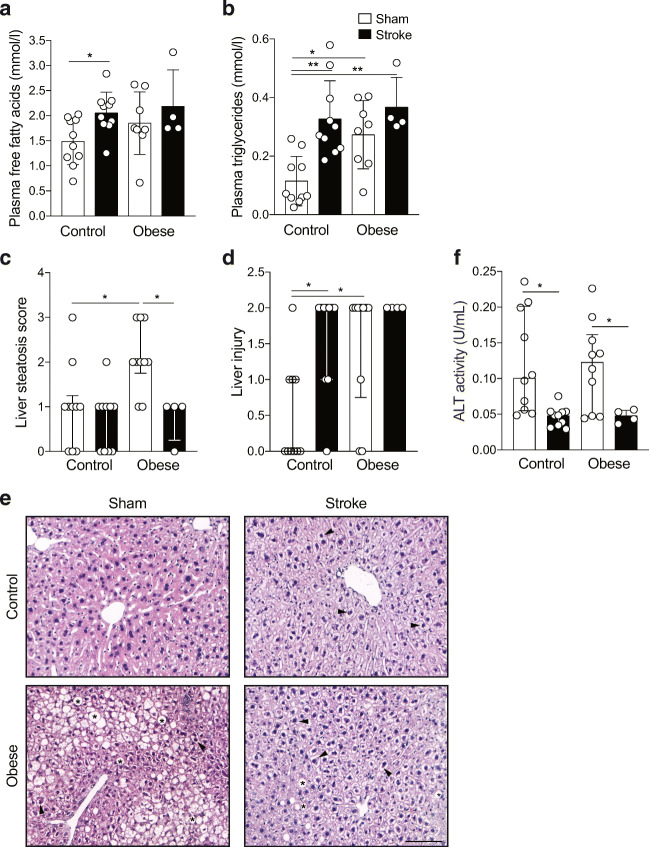
Stroke induces a prolonged increase in plasma lipids and change in liver pathology and function. At 60 day post-stroke, free fatty acids (**a**) and triglycerides (**b**) were assessed in the plasma. Liver steatosis and injury were assessed semi-quantitatively in tissue sections (**c** and **d**). Representative images of liver (**e**) showing steatosis (accumulation of fat) in obese mice (asterisks), and liver injury (ballooning of hepatocytes and/or pale to clear cytoplasm; arrow heads) in obese mice, and in control-fed mice. Scale bar 100 μm. Plasma ALT levels (**f**). In control mice, stroke led to an increase in plasma free fatty acids and triglycerides, a decrease in ALT, and caused a change in liver pathology/injury. Raised triglycerides, liver injury and lower ALT were observed in obese mice after sham or stroke surgery, while liver steatosis seen in obese sham mice was reduced after stroke. Data are presented as mean ± SD (**a**, **b**, **f**) or median and interquartile range (**c**, **d**). (*n* = 4–10). *p < 0.05 and **p < 0.01. Statistical analysis was performed using two-way ANOVA followed by Tukey’s post hoc multiple comparisons test for (**a**, **b**, **f**) and a non-parametric Kruskal-Wallis test followed by Dunn’s test for multiple comparisons for (**c**, **d**)

In mice fed a control diet, at 60 days after stroke, there was a significant increase in plasma free fatty acids and triglycerides (Fig. 6a, b). This effect was not seen in obese mice, though sham-operated obese mice had higher plasma triglycerides concentrations. In response to stroke in either control or obese mice, there was no significant correlation between weight loss and plasma free fatty acids (control *r*^2^ = 0.00002, obese *r*^2^ = 0.26) or triglycerides (control *r*^2^ = 0.05, obese *r*^2^ = 0.19).

Increases in liver triglycerides have previously been reported 24 h after stroke in mice [18]. Livers were therefore taken at day 60 and assessed histologically. Liver steatosis was observed in sham-operated obese mice, though this effect was lost in obese mice undergoing experimental stroke (Fig. 6c, e). Stroke had no effect on liver steatosis in control-fed mice. Hepatocyte injury was also measured using a scoring system, with damaged hepatocytes being identified by their partial or complete loss of cytoplasm, and condensed or swollen nuclei (Fig. 6d, e). Stroke induced significant hepatocyte damage in mice fed a control diet, though significant hepatocyte damage was already observed in sham-operated obese mice, and so stroke did not further increase hepatocyte damage in obese mice. ALT was measured as an indicator of liver function and was lower in both control and obese mice after a stroke compared to sham groups (Fig. 6f).

## Discussion

Here, we monitored the long-term metabolic and behavioural effects of stroke, and the impact of the co-morbidity obesity and demonstrate in control male mice long-lasting changes that could indicate a risk for future vascular health.

Stroke patients commonly develop depression and anxiety, which are barriers to recovery and rehabilitation [1, 3, 4]. Here, experimental stroke in mice induced prolonged changes in behaviours that were measures of sickness behaviour, depression and anxiety. Both nest building and burrowing behaviours were disrupted, two tests that exploit natural rodent behaviours, which could also be surrogate markers for activities of daily living. Burrowing behaviour has classically been employed as a measure of sickness behaviour in mouse models of systemic inflammation and prion disease [43, 44], but has also been suggested to measure attention and executive function [45]. Nest building, a complex goal-directed behaviour, has previously been used in models of schizophrenia to study negative symptoms including impaired attention [46, 47]. The reasons that patients develop depression are complex and poorly understood, though inflammation has been hypothesised to play a role [48]. Inflammation is well understood to drive sickness behaviour in rodents, which seems phenotypically similar to depression, involving decreased concentration, fatigue and anhedonia [49]. Prolonged anxiety was also seen after stroke, as mice spent more time at the sides of an open arena compared to the centre. It is unlikely that impairment in motor activity contributed solely to these behavioural changes (especially nest building and burrowing) after stroke as no changes in the distance moved or speed were observed in the open field test. Furthermore, we observed no deficits in nest building and burrowing at day 30 when there was still an impairment in sensorimotor function assessed using neuroscore. Some behaviours including burrowing have been proposed to be affected by repeated anaesthesia in healthy mice [50]. However, in the latter study, burrowing was assessed 30 min post-anaesthesia compared to at least 24 h here. Burrowing was not impaired in control mice after sham surgery, thus making it unlikely that anaesthesia contributed to the burrowing deficits in the present study. Collectively therefore, by using these tests, we have demonstrated that experimental stroke induced long-lasting changes in behaviour that were relevant to those seen in stroke patients.

Weight loss and malnutrition are frequently observed in patients with stroke and are associated with poor outcome [7–10]. It was originally thought that weight loss is due primarily to a reduction in lean mass, in particular muscle wasting (sarcopenia) [51] but recent work also demonstrates loss of fat mass [7]. Weight loss and reduction in muscle mass (examined ex vivo) have also been reported in rodents up to 7 days after experimental stroke [52–55]. These changes in muscle mass appear to be transient as no differences were observed between 2 and 14 weeks [56, 57]. Here, we comprehensively assessed the impact of stroke on body composition in vivo up to 49 days post-stroke, observing a prolonged reduction in body weight that was due to a loss of fat mass. Lean mass was also lost, but returned to pre-surgery levels by 21 days. The reason for this dramatic and prolonged weight loss after stroke is unclear and could involve several mechanisms, including reduced food intake and/or raised energy expenditure, possibly via the action of inflammatory mediators. Acute weight loss after experimental stroke can correlate positively with ischaemic damage [30, 58], although this correlation is not always observed [18]. Here, the degree of weight loss over 49 days did not directly correlate with initial damage at 48 h, suggesting that post-stroke weight loss may be caused by factors secondary to the initial injury, and develop during recovery. Experimental models of stroke involving permanent external carotid ligation have been proposed to cause feeding difficulties that result in weight loss [59, 60]. Although food intake is likely to be reduced in the initial few days after stroke, we observed that when weight loss was maximal (day 14), food intake was not different (in obese) or increased (in controls) compared to pre-surgery, which is in agreement with others showing no reduction in food intake 7 days after stroke in mice [54]. Furthermore, weight loss was significantly less in sham-operated mice (compared to stroke) in which external carotid ligation was also performed.

The long-term reduction in body fat we observed after experimental stroke was also accompanied by changes in lipid metabolism. This effect was most pronounced in non-obese animals, which showed increases in plasma free fatty acids and triglycerides. Non-obese animals also had higher plasma concentrations of resistin and adiponectin after stroke. It is unlikely that weight loss alone caused these changes as there was no correlation between the severity of weight loss and plasma levels of these factors. In addition, healthy weight loss is typically associated with a reduction in plasma free fatty acids and triglycerides. Experimental stroke has been previously shown to acutely affect peripheral lipid metabolism, potentially through the release of inflammatory mediators that can promote a catabolic state [18], and can also exacerbate atherosclerosis [61]. Together, these findings of lipid and adipokine disturbance here suggest that experimental stroke in non-obese mice resulted in long-lasting changes in metabolism, potentially originating in the adipose tissue. Changes in plasma adipokines and lipids in patients after stroke have been studied poorly, but a few studies report early increases in resistin and triglycerides [62–64]. Whether these factors in patients are high prior to stroke, and/or if they remain elevated more chronically is unknown. Stroke patients are at an increased risk of future vascular events, but it is not fully understood why [5, 6]. However, altered blood lipids [65], increased plasma resistin [20] and changes in adiponectin [66] have been reported as risk factors for stroke. Similarly, being underweight is a risk factor for stroke, and for poor stroke outcome [14–17]. Therefore, prolonged weight loss after stroke may result in a lipid and adipokine profile that increases the risk of further vascular events.

After stroke, non-obese animals also showed prolonged liver histological changes suggestive of liver injury. Previous work has indicated liver damage and hepatocyte apoptosis after stroke in high-fat fed rats, but only an acute time point (24 h) was studied [67]. Liver damage is also reported in other experimental models of injury including heart failure [68]. Within in the first 24 h after stroke in rodents, an increase in chemokine and cytokine expression is observed in the liver, which is accompanied by infiltration of neutrophils that could potentially initiate damage [18, 25, 67, 69]. An increase in liver triglyceride content is also seen 24 h after stroke in mice [18]. It is not known if liver triglyceride levels are chronically elevated in the present study, but this is unlikely as there was no increase in liver steatosis after stroke when assessed histologically. Liver damage is usually accompanied by an increase in plasma ALT, and raised plasma ALT have been reported between 3 and 24 h after stroke in rats [69, 70]. In contrast here, plasma ALT levels were lower 60 days after stroke compared to sham. However, low ALT blood levels have been shown to be a marker of frailty and be associated with increased risk of mortality and poorer long-term outcome in the middle-aged and elderly, and patients with ischaemic heart disease [71–76]. To our knowledge, chronic liver function has not been studied in ischaemic stroke patients, but liver disease is a co-morbidity and risk factor for stroke [77, 78] and lower ALT at admission is a predictor of poor outcome at 3 months [79]. Thus overall, these data suggest that experimental stroke induced a prolonged change in liver function, but whether this has a long-term consequence on health remains to be determined.

Obese rodents have greater infarcts after experimental stroke [25–27]. As the extent of ischaemic damage is the most important determinant of acute sensory and motor outcomes, it is also likely a key determinant of the longer-term outcomes studied here. Therefore, to model the clinical situation in which obese patients often do not have worse initial ischaemic injury and outcome [80, 81], we used different MCA occlusion times in our control and high-fat fed groups to match initial ischaemic damage. Indeed, there was no significant difference in infarct volume at 48 h or long-term survival up to 60 days. Furthermore, there was also no influence of obesity on the extent of odema, brain atrophy, physical impairment (when assessed by neuroscore) or depressive and anxiety-like symptoms. In contrast to the experimental studies, some clinical studies report an ‘obesity paradox’ where obese stroke patients have reduced mortality and morbidity. A hypothesised biological explanation for the ‘obesity paradox’ is that the greater metabolic reserves found in obese stroke patients protect them from severe weight loss, especially loss of lean tissue [28, 30]. In the present study, we found that obese mice had a greater reduction in fat mass after stroke compared to control mice. This could be partly due to food intake, as food intake was not increased after stroke (at day 14) in obese mice, and without a positive calorie balance the lost weight will not be regained. Enhanced weight loss in obese mice may also be driven by inflammatory cytokines that promote an enhanced anorexic response and/or raised metabolic rate. Increases in inflammatory cytokines are observed in obese mice after stroke in the plasma, liver and adipose tissue [18, 25]. Obese mice also show an enhanced anorectic response to lipopolysaccharide, which causes a greater and more prolonged reduction in food intake and body weight [82]. Besides losing more adipose weight after stroke, obese mice showed an altered long-term disturbance in lipids and adipokines compared to control mice, likely as obesity per se resulted in metabolic abnormalities (e.g. increased plasma lipids), which were similar to those found after stroke in control mice. Together, these data suggest that obesity did not offer protection against weight loss after experimental stroke or affect long-term behavioural changes.

In summary, stroke patients commonly develop secondary complications in the weeks and months after stroke that negatively affect their recovery and quality of life. However, these complications are not well studied pre-clinically, and their mechanisms are poorly understood. Here, we identified new behavioural tools to assess secondary complications of stroke such as depression. We also were the first to observe long-term effects of stroke on metabolic markers. Specifically, our data demonstrate that in both obese and control mice, lean mass is only transiently reduced after stroke, whereas we observed a long-term effect on fat mass. This reduction in fat mass in control mice was accompanied by changes in adipokines and lipids, and potential changes in liver function. Future work should determine if these findings in male mice are also seen in females, and how they translate to stroke patients. However, these data suggest stroke causes a lasting effect on metabolism that could contribute to the increased risk of recurrent vascular events seen in stroke patients.

## Electronic supplementary material

ESM 1 (DOCX 922 kb)
