# Supplementary material for: Stroke Induces Prolonged Changes in Lipid Metabolism, the Liver and Body Composition in Mice
Source: Transl Stroke Res. 2019 Dec 21;11(4):837–50. doi: 10.1007/s12975-019-00763-2 (PMC7340675; doi:10.1007/s12975-019-00763-2)
Supplement: Supplementary file 1 — (DOCX 922 kb) [file 12975_2019_763_MOESM1_ESM.docx]

**Supplementary material**

**Y-maze spontaneous alternation test**

To assess short-term working memory, the Y-maze spontaneous alternation test was performed as previously described [1]. Control and obese mice were assessed at day 0, and days 14 and 45 post-MCAO or sham surgery. Briefly, animals were placed in a white opaque Perspex maze with three arms (A, B and C) containing different visual cues and allowed to explore for 8 minutes before being returned to home cage. All tests were recorded by video and entries into arms were recorded (arm entries were defined as four paws crossing the threshold of a respective arm). Spontaneous alternation was defined as successive entry into three different arms. Subsequently, percent of correct alternation was calculated as the number of alternations divided by the number of arm entries minus two. The total number of moves was also recorded as an index of ambulatory activity All videos were scored blinded by an independent observer.

**Fig. S1** Stroke caused no impairment in memory. C57BL/6J fed a control or high-fat (obese) diet for 6 months underwent middle cerebral artery occlusion (MCAO; 30 min for control, 20 min for obese). At day 0, 14 and 45 working memory was assessed using the spontaneous alternation Y-maze test and was calculated as the % alternation (**a**). Locomotor activity and motivation to perform the test was assessed by number of entries (**b**). No difference in memory was observed at any time point after stroke but a decrease in the number of arm entries was seen after stroke in control mice at day 14 and an increase in obese mice at day 45. Data are presented as mean ± SD (*n* = 4-10). **p* < 0.05 control sham versus control stroke; ^##^*p* < 0.01 obese sham versus obese stroke. Statistical analysis was performed using a linear mixed effects models followed by Šidák-Holmes *post hoc* analysis.

1. Knight EM, Martins IV, Gümüsgöz S, Allan SM, Lawrence CB. High-fat diet-induced memory impairment in triple-transgenic Alzheimer's disease (3xTgAD) mice is independent of changes in amyloid and tau pathology. Neurobiology of Aging; 2014;35:1821–32.
